# Shifting daylength regimes associated with range shifts alter aphid‐parasitoid community dynamics

**DOI:** 10.1002/ece3.4401

**Published:** 2018-08-07

**Authors:** Rachel C. Kehoe, David Cruse, Dirk Sanders, Kevin J. Gaston, F. J. Frank van Veen

**Affiliations:** ^1^ College of Life and Environmental Sciences University of Exeter Penryn Cornwall UK; ^2^ Environment and Sustainability Institute University of Exeter Penryn Cornwall UK

**Keywords:** aphid, climate change, parasitoid, photoperiod, population dynamics, range expansion

## Abstract

With climate change leading to poleward range expansion of species, populations are exposed to new daylength regimes along latitudinal gradients. Daylength is a major factor affecting insect life cycles and activity patterns, so a range shift leading to new daylength regimes is likely to affect population dynamics and species interactions; however, the impact of daylength in isolation on ecological communities has not been studied so far. Here, we tested for the direct and indirect effects of two different daylengths on the dynamics of experimental multitrophic insect communities. We compared the community dynamics under “southern” summer conditions of 14.5‐hr daylight to “northern” summer conditions of 22‐hr daylight. We show that food web dynamics indeed respond to daylength with one aphid species (*Acyrthosiphon pisum*) reaching much lower population sizes at the northern daylength regime compared to under southern conditions. In contrast, in the same communities, another aphid species (*Megoura viciae*) reached higher population densities under northern conditions. This effect at the aphid level was driven by an indirect effect of daylength causing a change in competitive interaction strengths, with the different aphid species being more competitive at different daylength regimes. Additionally, increasing daylength also increased growth rates in *M. viciae* making it more competitive under summer long days. As such, the shift in daylength affected aphid population sizes by both direct and indirect effects, propagating through species interactions. However, contrary to expectations, parasitoids were not affected by daylength. Our results demonstrate that range expansion of whole communities due to climate change can indeed change interaction strengths between species within ecological communities with consequences for community dynamics. This study provides the first evidence of daylength affecting community dynamics, which could not be predicted from studying single species separately.

## 1 INTRODUCTION

Climate change has led to an increase in global temperatures (Hansen, Sato, Ruedy, Schmidt, & Lo, 2016), which is predicted to continue, with a projected increase in the mean global surface air temperature of 3.0°C by the end of the 21st Century (2071–2100), relative to the period between 1961 and 1990 (Flato et al., 2013; Houghton et al., 2001). The increase in global temperatures is causing a change in species ranges; a meta‐analysis with data consisting of 1,367 species from a wide variety of taxa showed poleward range shifts and expansions of between 12.2 and 91.1 km per decade (Chen, Hill, Ohlemüller, Roy, & Thomas, 2011).

While a poleward range shift allows populations to track climatic conditions, it also causes organisms to be exposed to other environmental conditions that do not match those within the original range. A key example of this is the daylength regime, with a poleward shift extending both summer days and winter nights and increasing the rate of daylength change in spring and autumn. Photoperiod drives many aspects of life history and activity patterns of temperate organisms (Beck, 2012; Vaartaja, 1959; Withrow, 1959) and thereby has the potential to affect population dynamics and species interactions.

Insects use photoperiod to a great degree as a cue to induce seasonal changes, for example in the induction of diapause (Adkisson, Bell, & Wellso, 1963; Ruberson, Bush, & Kring, 1991), as well as its termination (Tauber & Tauber, 1976), with these reactions dependent on geographic location (Lankinen, 1986), and in interaction with temperature (Liefting, Cosijn, & Ellers, 2017; Saunders, 1973). Some species have been shown to use photoperiod to influence egg morphology (Wardhaugh, 1977) while others use it to determine number of molts (Ingram & Jenner, 1976). Daylength has also been shown to have an impact on insect growth rate (Kamm, 1972), as well as development rate (Fisher, Higley, & Foster, 2015), fecundity (Nissinen, Pinto‐Zevallos, Jauhiainen, & Vänninen, 2017), and the regulation of insect seasonal development in nature (Abrams, Leimar, Nylin, & Wiklund, 1996; Danilevskii, 1965). However, there is currently a lack of studies investigating how photoperiod affects communities.

All these factors are likely to affect the interactions between species that drive ecological and evolutionary processes in ecosystems (Thompson, 1999) and are important for ecosystem stability (de Ruiter, Neutel, & Moore, 1995; Thébault & Fontaine, 2010). As species are interconnected within networks of interactions (Bukovinszky, van Veen, Jongema, & Dicke, 2008; van Veen, Memmott, & Godfray, 2006), a perturbation affecting one single species can therefore lead to community‐wide impacts, see Rosenblatt and Schmitz (2016) for a conceptual framework of the direct and indirect effects of climate change on a food web. For example, the harvesting of a single parasitoid species led to a community‐wide extinction cascade in a recent experiment, an effect that was transmitted indirectly via competition at the herbivore level (Sanders, Kehoe, & van Veen, 2015). Similarly, removing predators from an intertidal system led to extinctions of algae species through indirect interactions (Donohue et al., 2017). This demonstrates the importance of indirect as well as direct interactions for community stability. Intriguingly, it has also been shown that photoperiod disruption from artificial light at night can alter multitrophic insect community dynamics (Sanders, Kehoe, Tiley, et al., 2015).

Aphids are sap‐feeding herbivorous insects. Many are major pest species, especially when acting as vectors for plant viruses, causing critical damage to agricultural crops (Dedryver, Le Ralec, & Fabre, 2010). Their population and community dynamics have been studied extensively, including in the context of indirect species interactions (Hassell, 2000; Kaiser‐Bunbury & Müller, 2009; Müller & Godfray, 1999; Sanders, Sutter, & Veen, 2013; Sanders, Kehoe, & van Veen, 2015; Snyder & Ives, 2001) as well as climate change (Forrest, 2016). Aphids and aphid parasitoids are therefore an ideal model system to study population dynamics and species interactions in a community context as the system is very tractable and the generation times are short (Sanders, Kehoe, Tiley, et al., 2015), allowing for the observation of parasitoid‐host interactions across a multigenerational time frame.

**Figure 1:**
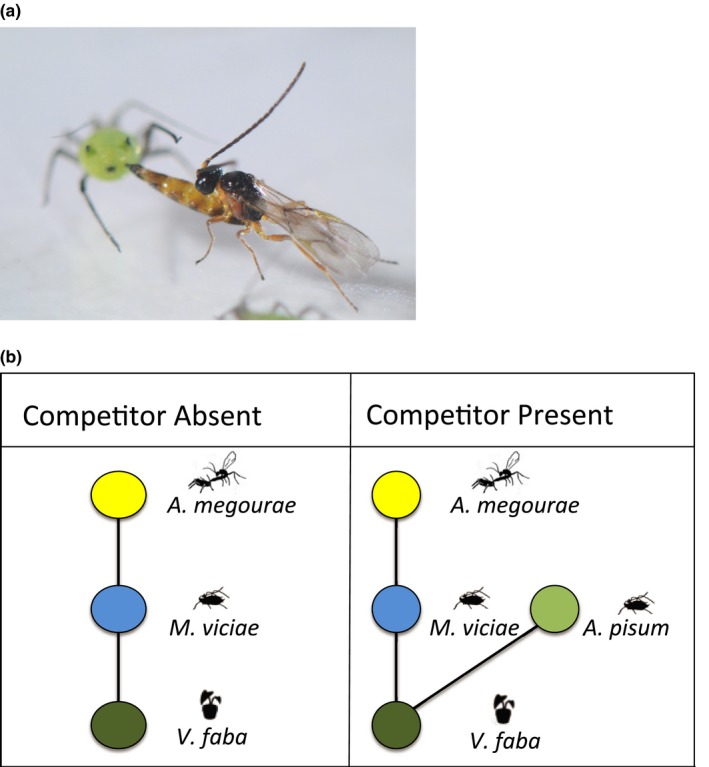
(a) *Aphidius megourae* attacking *Megoura viciae*. (b) Food web structure for experimental “Competitor Absent” and “Competitor Present” communities [Colour figure can be viewed at http://wileyonlinelibrary.com]

Here, we study for the first time, the effects of daylength on the dynamics of multitrophic communities, while keeping other factors such as temperature and the rate of change in daylength constant to test for the impact of short and long daylength in isolation. We focus in particular on the effects during summer conditions, when populations of aphids reach their greatest pest potential. In our experiments, we used a simple host‐parasitoid community consisting of two aphid species that compete for a single host plant species and a parasitoid that attacks one of the aphid species (Figure 1b). We hypothesized that longer daylength, associated with a poleward range shift, would increase the attack rate by the diurnal parasitoid and that this would (a) negatively affect the host aphid population and, through reduced interspecific competition, (b) positively affect the other aphid species. We show that while the host‐parasitoid interaction was not affected by daylength, we discovered that the competitive strength of the two aphid species changed with daylength resulting in higher *Megoura viciae* abundance under long days.

## 2 MATERIALS AND METHODS

### 2.1 Study system

The study system consisted of broad bean plants (*Vicia faba*, L., var. the Sutton), which supported two aphid species, *M. viciae* (Buckton) and *Acyrthosiphon pisum* (Haliday), and the parasitoid *Aphidius megourae* attacking the aphid *M. viciae* (Figure 1a).

### 2.2 Food web experiment

We used eight climate chambers (Percival Model 1‐30vl) programmed to constant 22°C and 75% humidity. The temperature was kept constant so as to enable the separation of daylength from any confounding impact of temperature, which has been shown to be linked to photoperiod, see Fischer et al. (2012). To test for the effect of daylength on aphid‐parasitoid communities, four chambers produced a day–night cycle of 14.5–9.5 hr (Southern) (depicting Marseille, France, 43°N, average daylength for the 9 weeks either side of the summer solstice), while the other four units produced a 22–2‐hr day–night cycle (Northern) (replicating Mosjoen, Norway, 65°N for the same time period). These locations were used to provide two distinct conditions for summer days, and daylength was kept constant in order to test for daylength per se and not the rate of daylength change. The intensity of the light within the incubators during “daylight” hours was recorded at 4,239 lux, equivalent to a typical overcast day (Gaston, Bennie, Davies, & Hopkins, 2013). We established two different communities with the aphid *A. pisum* either included “Competitor Present” or excluded “Competitor Absent.” This extended community allows for resource competition (Holt, 1977) between the two aphids and the potential for indirect interactions among the insects. Within each chamber were four cages, two consisting of the “Competitor Absent” community, with the other two consisting of the “Competitor Present” community, thus giving four treatments, each replicated eight times (see Figure 1). These cages were 35 cm × 24 cm × 20 cm and were constructed of untreated timber and thrip net with a mesh size of 0.29 mm × 0.8 mm, each with four 15 cm diameter pots containing a single broad bean plant in Melcourt All‐purpose Peat Free Compost.

All insects used in this experiment were taken from laboratory stock cultures, reared on broad bean plants at a temperature of 18°C and at a 16:8 day:night regime, for a number of years, and were kept at low insect densities. We tested for a difference in the growth rate of aphids under different daylength regimes from these stock cultures to those reared for three generations at the short daylength regime and found no difference for growth under short and long days (Supporting Information Figure S1). There was no impact of the origin of either species of aphids on their growth rate (*M. viciae* GLM Offspring number ~ Origin, mean = 13.371, *T* = 0.499, *p* = 0.621, *A. pisum* GLM Offspring number ~ Origin, mean = 18.361, *T* = −1.234, *p* = 0.226).

To establish the replicate insect communities, in week 1, five parthenogenetically reproducing adults of each aphid species (dependent on the treatment) were placed onto four 2‐week old broad bean plants and set into the climate chambers. At week 4, once aphid numbers had grown large enough to support an additional trophic level, two female, mated parasitoids of *A. megourae* were introduced to each cage with a further two added at week 5. This double introduction allowed for continuous production of parasitoids throughout the experiment. The numbers of both aphids and parasitoid mummies, the latter depicting a successful attack on aphids, were recorded. This count was repeated weekly over a 9‐week period, equivalent to 9–10 aphid generations. Plants were watered every second day throughout the experiment, with the oldest plants in each cage being replaced weekly with 2‐week‐old plants in order to ensure a continual food source for the aphids, while keeping all organisms in the cage. This method has been shown in Sanders et al. (2013). The cages were rotated within and between incubators of the same treatments weekly in a block design to account for a potential incubator bias.

### 2.3 Competition experiment

In order to explain the effects of the main experiment, we set up an additional competition experiment using three aphid combinations; *A. pisum* only, *M. viciae* only and a combination of two species. Two adult aphids of each species, depending on the species combination, were placed onto a 2‐week‐old broad bean plant over which a breathable bag was placed and secured with a rubber band. These were then placed into an incubator at photoperiods of either 14.5:9.5 or 22:2, at 22°C. The number of aphids was counted weekly for 3 weeks. Each treatment was replicated 10 times.

### 2.4 Statistical analysis

#### 2.4.1 Food web experiment

Aspects of aphid and parasitoid population dynamics were analyzed using generalized linear models (GLM) with daylength treatment and community as explanatory variables. We used the following response variables:

1. Log‐transformed cumulative abundance (for each species, the total number of individuals for each cage over the length of the experiment), with Gaussian error structure.
2. Peak abundance. This is an ecologically important population measure for pest insects. This was measured as the maximum population size of each species at any point during the experiment and was analyzed using a GLM with Gaussian error structure. The data for *M. viciae* and *A. pisum* were normally distributed, whereas data for *A. megourae* were log transformed, to improve fit to a normal distribution.
3. Parasitism rate (proportion of hosts parasitised). This was analyzed using a GLM with a quasibinomial error structure. The response variable included the parasitized and nonparasitized aphid numbers per cage (using “cbind” in R).
4. Aphid population growth rate. This was analyzed using a GLM with a quasiPoisson error structure. Growth rate was calculated as daily increase in aphid number per cage between week 2 and week 4 (week 4 number − week 2 number, then divided by 14). These points were chosen as by that time there was no impact of parasitism on aphid numbers before week 4.

#### 2.4.2 Competition experiment

The impact of treatment (four treatments: 14.5 single, 14.5 competitor present, 22 single, 22 competitor present) on aphid cumulative numbers were tested using linear models based on generalized least squares (errors are allowed to have unequal variances) provided by the nlme package (Pinheiro et al., 2017). We used VarIdent to account for variance heterogeneity in effect sizes between treatment groups. This test was replicated for both *A. pisum* and *M. viciae*. A Tukey's comparison was then used as a post hoc test for between treatment contrasts.

Throughout, best fitting models were chosen using AIC model selection (Akaike, 1998). Models for all analyses were visually checked for homoscedasticity and normality of the residuals, and all fulfilled the assumptions. All statistical analyses were computed using R version 3.2.1 (R Development Core Team, 2015).

## 3 RESULTS

### 3.1 Cumulative aphid abundance

**Figure 2:**
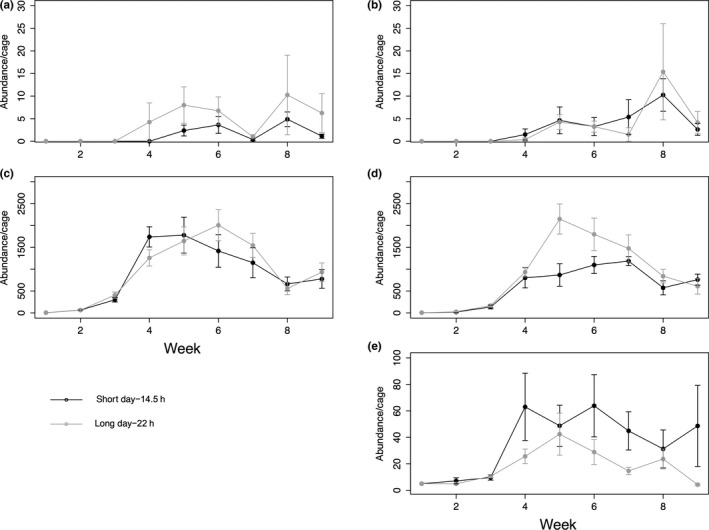
Population dynamics of all species. Sub plots (a and b) depict the parasitoid *Aphidius megourae*, (c and d) the aphid *Megoura viciae* and (e) the aphid *Acyrthosiphon pisum*. “a” and “c” show dynamics of the “Competitor Absent” community, while “b,” “d” and “e” depict the “Competitor Present” community. Black lines show Southern treatments, with gray lines showing Northern treatments. Error bars indicate standard error

The aphid *M. viciae* was not affected by daylength in the absence of the competitor *A. pisum*, but in its presence *M. viciae* densities were 32% higher in the Northern compared to the Southern treatment with *A. pisum* present (Figure 2, GLM Community × Daylength *t* = −2.09 (1, 28), *p* = 0.0495).

*Acyrthosiphon pisum* was negatively affected by a longer daylength, with populations 50% smaller compared to the Southern treatment (*t* = 2.21 (1, 14), *p* = 0.03).

Neither community nor daylength affected the abundance of the parasitoid *A. megourae* (Daylength GLM *t* = −0.715 (1, 30), *p* = 0.481) (Community GLM *t* = 0.78 (1, 29) *p* = 0.44), see Figure 2.

### 3.2 Peak aphid abundance

**Figure 3:**
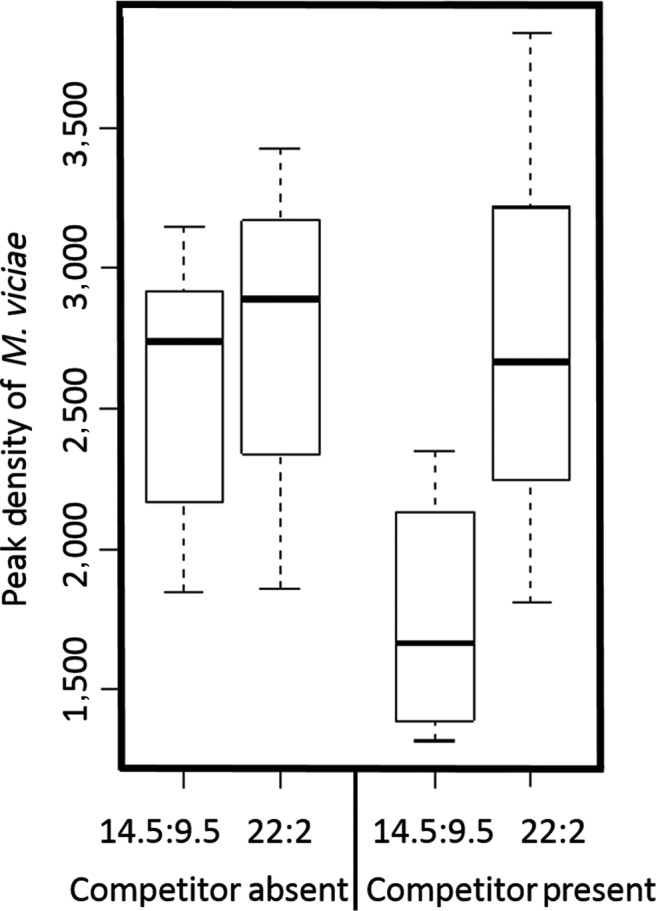
Peak density of *Megoura viciae*, divided into “Competitor Absent” community and “Competitor Present” community, and then subdivided into long and short daylength treatments

Peak abundances of *M. viciae* were not affected by daylength in the absence of the competitor but in the presence of the competitor, the Northern treatment lead to 56% higher abundances than the Southern Treatment with *A. pisum* present (GLM Community × Daylength *t *= −3.32 (1, 28) *p* = 0.003). The peak densities of both the aphid *A. pisum* (Treatment GLM *t* = 1.52 (1, 16), *p* = 0.15) and the parasitoid *A. megourae* (Treatment *t* = −0.53 (1, 30), *p* = 0.6, community *t* = 1.12 (1, 29), *p* = 0.27) were not affected by daylength, see Figure 3.

### 3.3 Parasitism rate

Parasitism rate of the aphid *M. viciae* by the parasitoid *A. megourae* was not affected by daylength or the presence of competitor (Treatment [Daylength, Community] GLM *t* = −0.56 (1, 30), *p* = 0.6).

### 3.4 Aphid population growth rate

The population growth rate of *M. viciae* was reduced by 72% by the presence of the competitor, *A. pisum* (*t* = −2.90 (1, 29) *p* = 0.007), with no effect of daylength (*t* = −0.85 (1, 30) *p* = 0.40), or interaction between daylength and community (*t *= 1.352 (1, 31), *p* = 0.187). Daylength regime did not affect the population growth rate of *A. pisum* (*t* = −1.51 (1, 14), *p* = 0.15).

### 3.5 Competition experiment

**Figure 4:**
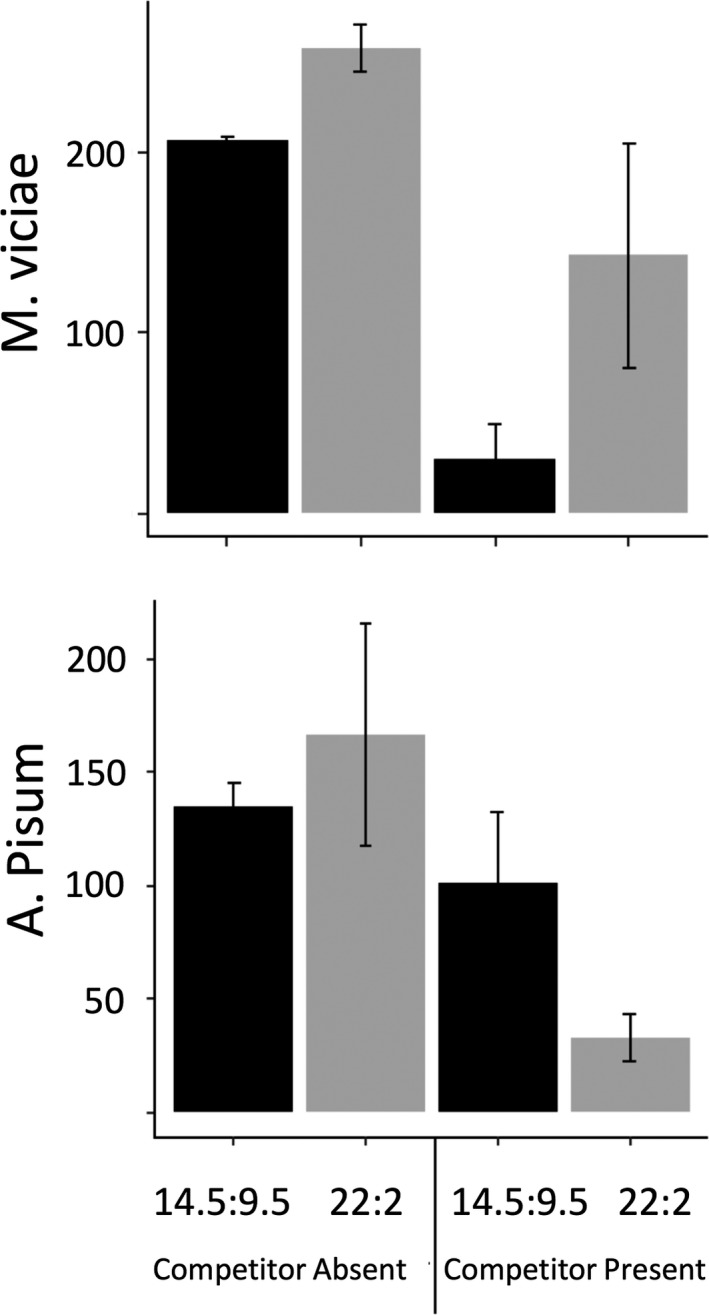
The mean cumulative density and standard error of (a) *Megoura viciae* and (b) *Acyrthosiphon pisum* in long (22:2) and short (14.5:9.5) daylengths, with and without a competitor. Black bars depict short daylengths, and gray long daylengths

In an additional experiment, we tested whether aphid growth would be affected by the interplay between daylength treatment and competition between aphids in the absence of parasitoids. *Megoura viciae* numbers were indeed reduced when competing with *A. pisum* under short daylength but not long days (see Figure 4). *Megoura viciae* abundance declined by 86% in the presence of *A. pisum* under short day conditions (*z* = −5.35 *p* < 0.001). Interestingly, the opposite pattern was observed for *A. pisum*, with its densities being strongly negatively affected with a reduction by 81% in the presence of *M. viciae* under long day condition (see Figure 4, *z *= −2.65 *p* = 0.036). *Megoura viciae* densities were also higher under 22 than 14.5 daylength indicating that in isolation *M. viciae* grows better under longer (Northern) summer days (see Figure 4, *t* = 4.03 (1, 10), *p* = 0.002).

## 4 DISCUSSION

We expected that longer daylength would increase parasitoid attack rate, which would in turn (a) negatively affect the host aphid population and, through reduced interspecific competition, (b) positively affect the other aphid species. We did not observe this but instead found a decrease in cumulative abundance of the aphid *A. pisum* under Northern conditions, coupled with an increase in cumulative and peak abundances of the aphid *M. viciae*. However, *M. viciae* did not respond to daylength when it was the only aphid species present in the food web experiment. This shows that an increase in summer daylength, associated with a poleward range shift, has an indirect positive impact on one pest species due to reduced competition from another that is negatively affected by increased daylength. Interestingly, the competition experiment demonstrated that the competitive dominance between the two aphids species switched with daylength. *Megoura viciae* is the dominant competitor under long days while it suffers more from competition with *A. pisum* under short days. This explains the outcome in the food web experiment, with *M. viciae* profiting from longer days under Northern conditions. This effect appears to be driven by different growth rates of *M. viciae* under the different daylength regimes as shown in the competition experiment. This effect was not visible in the food web experiment, maybe due to the more complex setting of the experiment.

One might expect that the population growth of, essentially sessile, sap‐feeding insects, such as aphids, will mostly be affected by changes in photosynthesis of their host plant which will determine resource availability, with aphid reproduction rate depending on the growth stage of its host plant (Watt, 1979), as well as the plant's degree of water stress (Simpson, Jackson, & Grace, 2012). Photosynthesis is highly dependent on photoperiod, with photosynthetic activity increasing with increasing photoperiod (Bauerle et al., 2012). The increase in growth rate for *M. viciae* in the competition experiment supports this effect in our experiments. However, this evidence that longer summer days had positive effects either on the early population growth rate of aphids was not found in the food web experiment. In fact, a negative effect was observed for *A. pisum* cumulative abundance, reflecting sustained differences between the treatments over at least three generations (Figure 2e). Photoperiod has been shown to affect individual growth rate and body size for a number of insects, a response that may or may not be adaptive (Gotthard, Nylin, & Wiklund, 1999; Margraf, Gotthard, & Rahier, 2003; Shama & Robinson, 2006) and we suggest that *A. pisum* and *M. viciae* were indeed affected by daylength but with very different outcomes, which is intriguing because the two species are ecologically and phylogenetically similar.

Our prediction that longer days would lead to increased top‐down control of aphids by parasitoids due to extended activity patterns had a number of underlying assumptions. First of these is that parasitoids are time‐limited rather than egg‐limited, or, in other words, that the number of hosts a female parasitoid parasitises is limited by the number of hosts that she encounters (Henri & Van Veen, 2011). It is not unlikely that in the confines of our experimental cages, with high densities of aphids, the female parasitoids encountered a sufficient number of hosts for all their eggs even in the shorter day. Further research is required under realistic field conditions in which host encounter rates will be lower to test the effect of changes in photoperiod on parasitoid efficiency. Our second assumption was that increased parasitoid attack rate would lead to increased parasitoid population growth and increased parasitism rate of the host aphid. It is, however, possible that higher attack rates lead to reduced parasitoid lifespan (Werner & Anholt, 1993) so that there is overall little net effect on the parasitoid population growth. It should also be noted that the parasitoid populations in the experiment remained relatively low despite the abundance of hosts. This indicates that larval survival of the parasitoids may have been low due to the competitive inferiority of parasitized aphids compared to unparasitised aphids under crowded conditions (Ives & Settle, 1996). This may have further weakened the effect of a change in attack rate on the numerical response of the aphid population. Again, this effect is likely to be less important under natural conditions because of nonuniform host distributions and therefore greater variation in intraspecific competition in the population.

Another mechanism by which the parasitoid *A. megourae* might have impacted upon the community is through their reluctance to parasitise aphids in unlit periods (Sanders, Kehoe, Cruse, van Veen, & Gaston, 2018), as well as the disruptive effect of nonhosts in the community reducing parasitism rate (Kehoe et al., 2016). Both of these mechanisms, however, do not explain the direction of the interaction, and as such we can conclude that these effects were overwhelmed by bottom‐up effects.

Understanding of how ecosystems do and will respond to climate change and associated range expansion of species needs to take into account that shifts in day–night regimes can trigger significant changes in species interactions. Our study was limited to summer conditions and it is likely that a year‐round perspective that includes key life‐cycle stages such as diapause would reveal further effects on insect community dynamics. The responses of agricultural pests to climate change remains one of the main unknown factors in the ability to predict crop productivity under future climate scenarios (Gornall et al., 2010; War, Taggar, War, & Hussain, 2016), although see (Gebauer, Hemerik, & Meyhöfer, 2015). With crop plants already grown outside of their natural range, the range expansion of any insects using them as a host plant will be instantaneous, as they do not require the expansion of their host plant range. As our study shows, species responses should not be studied in isolation but should be considered in the context of communities of interacting species, taking into account the change in abiotic factors such as photoperiod as well as evolutionary processes associated with poleward range shifts and expansions.

## CONFLICT OF INTEREST

None declared.

## AUTHORS’ CONTRIBUTIONS

FvV and KG conceived the ideas; FvV, KG, and DS designed methodology; DC collected the data; RK analyzed the data; RK, DS, and FvV led the writing of the manuscript. All authors contributed critically to the drafts and gave final approval for publication.

## DATA ACCESSIBILITY

Data available from https://figshare.com/s/bdd2951343e341820014.
