# Supplementary figures and images for: Shifting daylength regimes associated with range shifts alter aphid‐parasitoid community dynamics
Source: Ecol Evol. 2018 Aug 7;8(17):8761–9. doi: 10.1002/ece3.4401 (PMC6157684; doi:10.1002/ece3.4401)

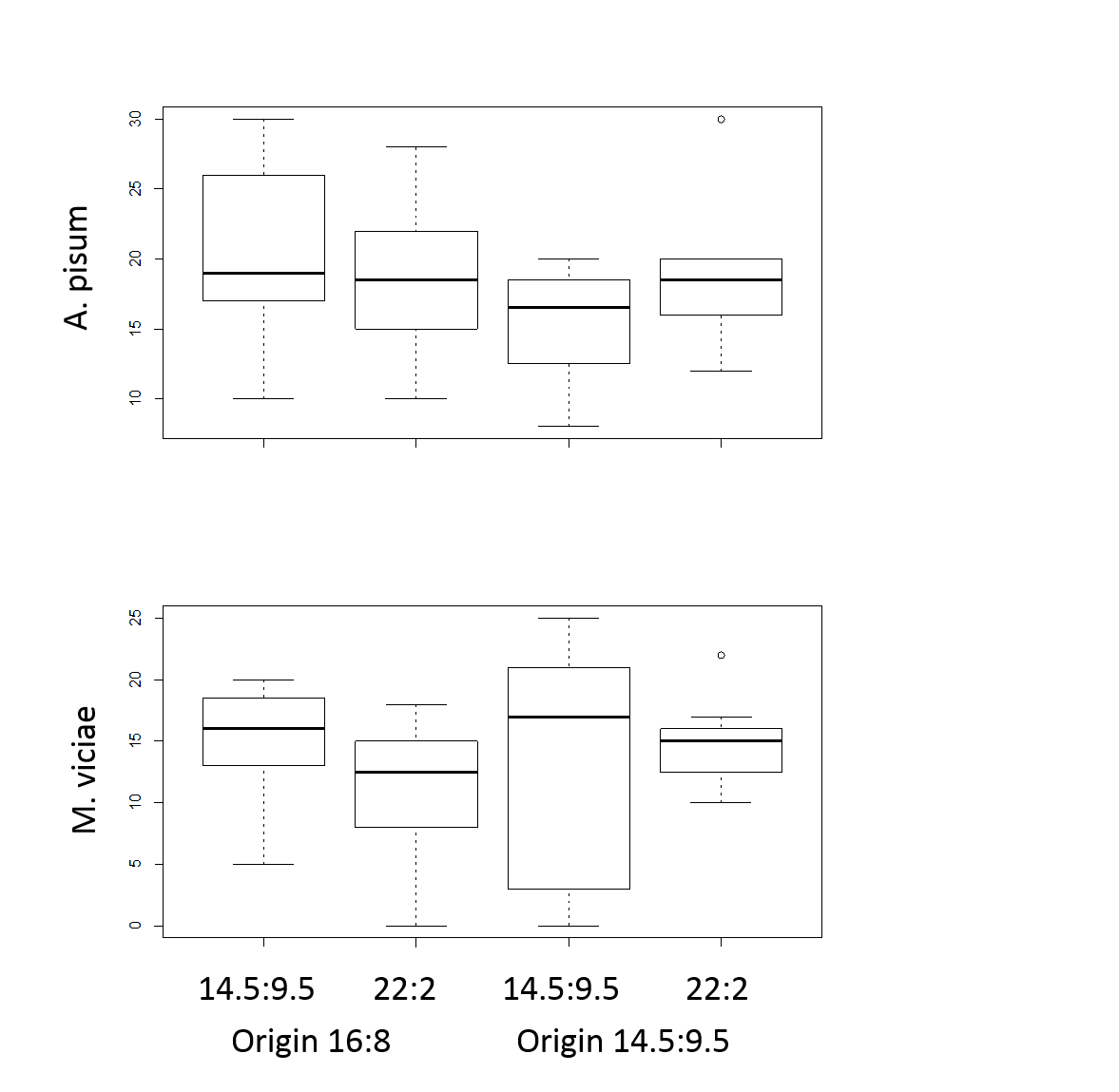

Supplement: Supplementary file 1 [file ECE3-8-8761-s001.tif]
